# Supplementary material for: Sensorimotor transformation of number in the primate parietal cortex
Source: Nat Commun. 2026 May 11;17:4227. doi: 10.1038/s41467-026-73037-9 (PMC13161402; doi:10.1038/s41467-026-73037-9)
Supplement: Supplementary file 1 — Supplementary Information [file 41467_2026_73037_MOESM1_ESM.pdf]

Sensorimotor transformation of number in the primate parietal cortex

Laura E. Seidler[1], Stephanie Westendorff[1] & Andreas Nieder[1]*

[1] *Animal Physiology Unit, Institute of Neurobiology, Auf der Morgenstelle 28, University of Tübingen, 72076 Tübingen, Germany*

* Corresponding author: andreas.nieder@uni-tuebingen.de;

ORCID:

Andreas Nieder: 0000-0001-6381-0375

Supplementary Information

Supplementary Figures 1–4

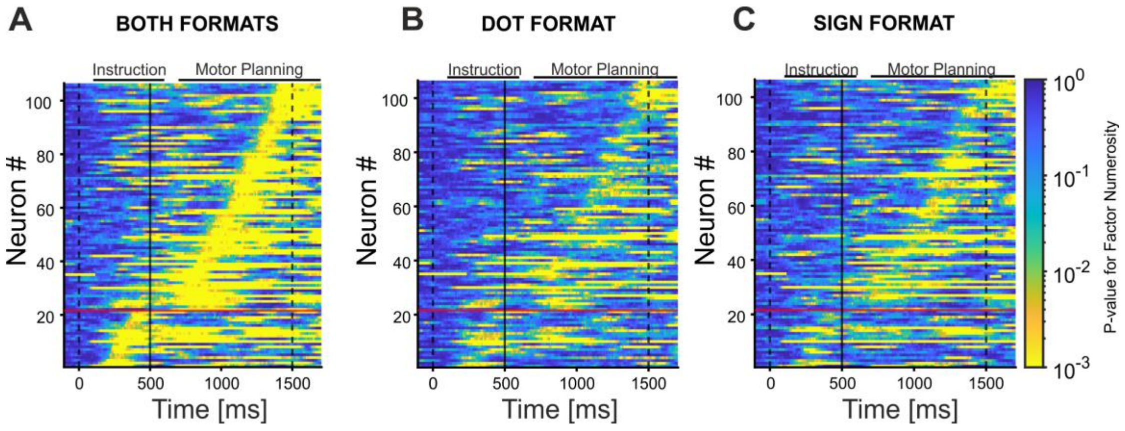

Supplementary Fig. S1: Number-selective time intervals for different format conditions.

Selectivity intervals of single neurons during the instruction stimulus phase (0-500 ms) and motor planning phase (500-1500 ms). Each line represents the activity of one neuron (n = 106), with surface color indicating the p-value of selectivity. The thick solid lines on top of the surface plot delineates the sliding-window ANOVA analysis interval for the respective phases (accounting for neuronal response latencies). **A)** Both dot and sign formats. **B)** Only dot format. **C)** Only sign format. (Source data are provided as a Source Data file).

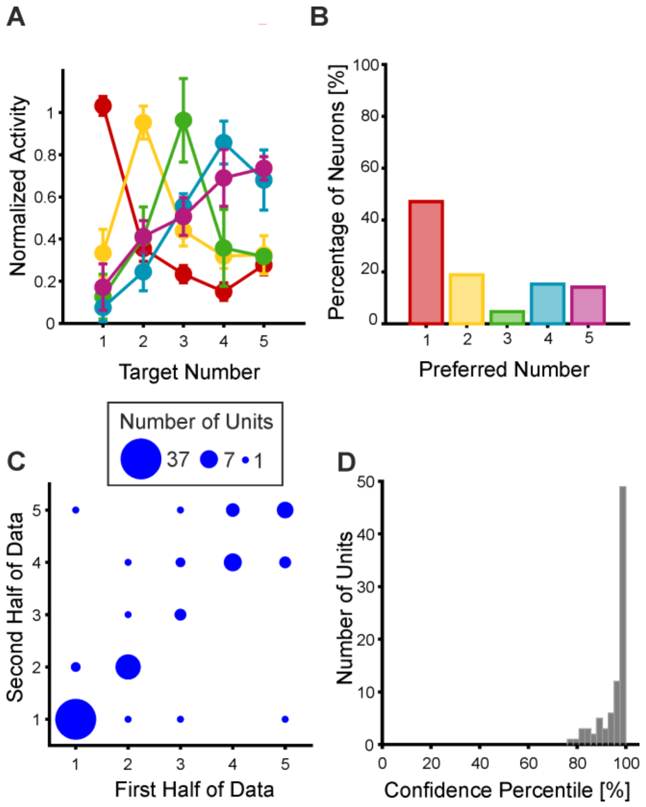

Supplementary Fig. S2: Split-half cross-validation of tuning curves.

A) Normalized average tuning curves, when using half of the data to determine the preferred number, and the other half of the data to calculate the tuning curves. Trials (even versus odd) for each neuron were split into balanced training and test sets (50/50). Preferred numerosity was defined from the training set, and normalized tuning curves were computed on the independent test sets. Error bars represent the SEM across neurons, (*n* = 40, 16, 4, 13, 12 neurons for preferred numerical values 1–5, respectively).

B) Distribution of preferred numerical values of test sets.

C) Preferred numbers in the training set are correlated with preferred numbers in the test sets. Median correlation coefficient = 0.91 (IQR = 0.77-0.97). (Pearson's r = 0.85 p = 8.48*10^{-25}).

D) Confidence percentile position of the neurons' real cross-validation tuning correlation coefficients relative to the neurons' chance correlation coefficient distribution with shuffled data. Median confidence percentile = 98.17 % (IQR = 93.71 % - 99.47 %). (Source data are provided as a Source Data file).

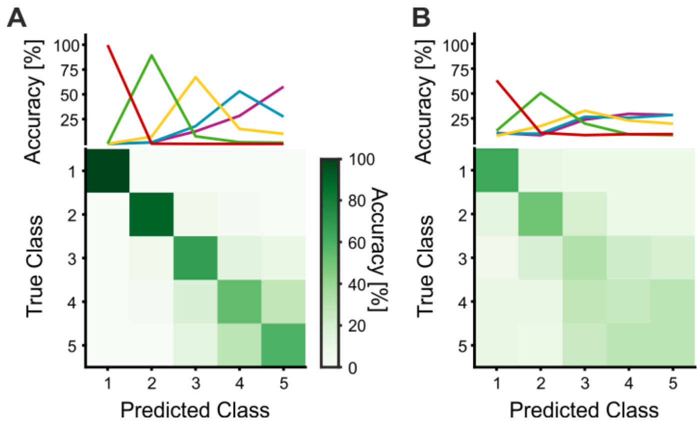

Supplementary Fig. S3: ***Classifier decoding analysis for different cell populations.***

A) Performance of an SVM classifier decoding target numerosities (1 to 5) from the firing rates of all numerosity-selective VIP neurons during the motor planning period. Top: Accuracy curves for each target numerosity, color-coded. Bottom: Corresponding confusion matrix showing predicted versus instructed number of movements. The main diagonal indicates correct classifications. (*n* = 85 neurons, (averaged across 10-fold cross-validation and 1000 resamples). **B)** Same as in A, but for the population of unselective neurons. (*n* = 161 neurons) (Source data are provided as a Source Data file).

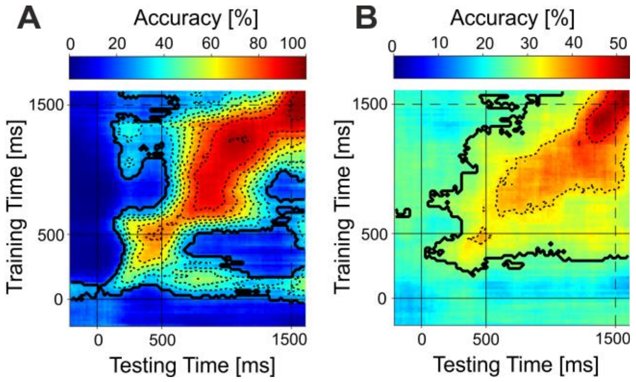

Supplementary Fig. S4: ***Cross-temporal classification accuracy for neurons predicting specific labels.***

A) Neurons predicting numerosity 1 trials. **B)** Neurons predicting numerosity 2 to 5 trials. Mean accuracy is color-coded in a 2D matrix, with training times on the x-axis and testing times on the y-axis. Time 0 ms marks instruction stimulus onset (solid black line), and 500 ms marks the start of the motor planning period (dashed line), which lasts until 1500 ms. Significant accuracy clusters at 25% accuracy are outlined in black (cluster permutation test). Dashed contour lines indicate accuracy levels from 35% to 65% in steps of 10%. (*n* = 246 neurons). (Source data are provided as a Source Data file).
